# Rapid Increase in Suspected SARS-CoV-2 Reinfections, Clark County, Nevada, USA, December 2021

**DOI:** 10.3201/eid2810.221045

**Published:** 2022-10

**Authors:** Jeanne Ruff, Ying Zhang, Matthew Kappel, Sfurti Rathi, Kellie Watkins, Lei Zhang, Cassius Lockett

**Affiliations:** Centers for Disease Control and Prevention, Atlanta, Georgia, USA (J. Ruff);; Southern Nevada Health District, Las Vegas, Nevada, USA (Y. Zhang, M. Kappel, S. Rathi, K. Watkins, L. Zhang, C. Lockett)

**Keywords:** COVID-19, respiratory infections, severe acute respiratory syndrome coronavirus 2, SARS-CoV-2, SARS, coronavirus disease, zoonoses, viruses, coronavirus, reinfection, Omicron variant, Nevada, United States

## Abstract

Genetic differences between SARS-CoV-2 variants raise concerns about reinfection. Public health authorities monitored the incidence of suspected reinfection in Clark County, Nevada, USA, during March 2020–March 2022. Suspected reinfections, defined as a second positive PCR test collected >90 days after an initial positive test, were monitored through an electronic disease surveillance system. We calculated the proportion of all new cases per week that were suspected reinfections and rates per 1,000 previously infected persons by demographic groups. The rate of suspected reinfection remained <2.7% until December 2021, then increased to ≈11%, corresponding with local Omicron variant detection. Reinfection rates were higher among adults 18–50 years of age, women, and minority groups, especially persons identifying as American Indian/Alaska Native. Suspected reinfection became more common in Clark County after introduction of the Omicron variant, and some demographic groups are disproportionately affected. Public health surveillance could clarify the SARS-CoV-2 reinfection burden in communities.

The emergence of new SARS-CoV-2 variants with antibody-evading mutations raises concerns about variable levels of protection against infection after prior infection or vaccination (*1*). The Omicron variant is genetically divergent from previous variants, exacerbating these concerns (*1*). Reinfection with SARS-CoV-2 after previous infection has been demonstrated through a comparison of viral genomes collected from the same person (*2*). However, without genomic sequencing, reinfection can be difficult to distinguish from prolonged viral shedding. Available evidence suggests an interval of at least 90 days between positive tests is more likely to indicate reinfection than prolonged viral shedding (*3*).

Public health authorities at the Southern Nevada Health District (SNHD) conducted surveillance of suspected reinfections in Clark County, Nevada, USA, to determine whether previously infected persons were protected against reinfection with new variants and to estimate the proportion of COVID-19 cases that occurred among persons with previous SARS-CoV-2 infections. SNHD also compared rates of suspected reinfection between demographic groups to characterize the groups most affected by suspected reinfection in Clark County and determine whether any groups were disproportionately affected. We report findings from surveillance of suspected reinfection with SARS-CoV-2 and rates of suspected reinfection among demographic groups in Clark County during March 2020–March 2022.

## Methods

Health care providers, medical facilities, laboratories, and other out-of-state health departments report positive SARS-CoV-2 PCR test results for residents of Clark County to SNHD. These results are collected in an electronic disease surveillance system. We calculated intervals between the specimen collection date from each person’s initial positive PCR test and subsequent positive PCR tests. We considered a subsequent positive PCR test with specimen collection >90 days after specimen collection of the initial positive PCR test to be a suspected reinfection (*3*). Repeat positive PCR tests with specimen collection dates <90 days after the specimen collection of an initial positive PCR test were not considered suspected reinfections and were excluded from the analysis. 

We calculated the proportion of new cases per week that were suspected reinfections by dividing the number of suspected reinfections by all new PCR-identified cases during the same week. We also identified suspected third infections, defined as a third positive PCR test collected at least 90 days after specimen collection of a second positive PCR test that met the above definition of suspected reinfection (*3*). Because of a small number of suspected third infections, we did not calculate the proportion of cases for suspected third infections. 

We gathered demographic information from case investigation data. We calculated the rate of suspected reinfections per 1,000 previously infected persons by age group, sex, and race/ethnicity. We calculated odds ratio (OR), 95% CI, and p value by using logistic regression and performed all analyses in SAS version 9.4 (SAS Institute Inc., https://www.sas.com). This activity was reviewed by the US Centers for Disease Control and Prevention (CDC) and was conducted consistent with CDC policy and applicable federal law, including the following Code of Federal Regulations (CFR) and US Code (USC): 45 CFR part 46; 21 CFR part 56; 42 USC Section 241(d); 5 USC Section 552a; 44 USC Section 3501 et seq. 

## Results

**Figure 1:**
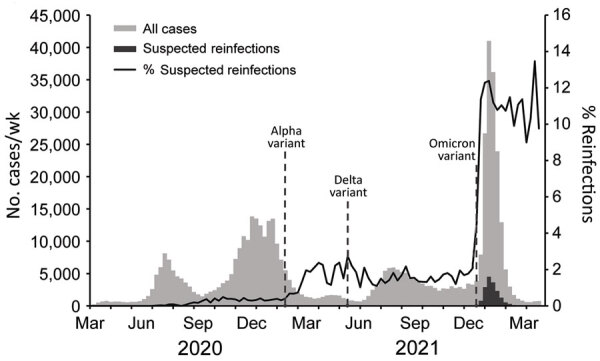
SARS-CoV-2 cases and suspected reinfections, Clark County, Nevada, USA, March 2020–March 2022. Dotted lines show timeframe for identification of Alpha, Delta, and Omicron SARS-CoV-2 variants in Clark County. New cases were defined as a first positive SARS-CoV-2 PCR test for a person. Suspected reinfections were defined as a second positive SARS-CoV-2 PCR test collected >90 days after a person’s first positive PCR test.

During March 2020–April 2, 2022, SNHD identified 19,589 suspected reinfections in Clark County; reinfections began occurring in June 2020. The incidence of suspected reinfection remained <0.5% of new cases until February 2021 (Figure 1). During the last week of February 2021, the incidence increased to ≈2% of all new cases. This increase in suspected reinfections occurred after the Alpha variant (B.1.1.7) was detected in Clark County in late January 2021 (*4*). During March 2021–November 2021, incidence of suspected reinfection remained at 1%–2.7% of cases, even after the Delta variant was detected in Clark County in May 2021 (*5*). In December 2021, we observed a rapid increase in the incidence of suspected reinfection, from 2% during the week of December 5–11 to 11% during the week of December 19–25. This rapid increase corresponded with an unprecedented rise in first-time infections and detection of the Omicron variant, which was reported in Clark County on December 14, 2021 (*6*). Although the weekly number of both suspected reinfections and first-time infections decreased substantially after a peak during the first week of January 2022, the proportion of suspected reinfection cases remained elevated, near 11%, through March 2022.

The rate of suspected reinfection was highest among adults 18–49 years of age; rates were 49/1,000 cases for persons 18–24 years of age and 46/1,000 cases for persons 25–49 years of age (Table). The odds that children <5 years of age were reinfected (15/1,000 cases; OR 0.30, 95% CI 0.26–0.35) was 70% lower than that for adults 18–24 years of age. Adults >65 years of age had 58% lower odds of suspected reinfection (rate 21/1,000 cases; OR 0.42, 95% CI 0.40–0.45) than adults 18–24 years of age. Women had a higher suspected reinfection rate (44/1,000 cases) than men (33/1,000 cases) and had 36% higher odds of suspected reinfection compared with men (OR 1.36, 95% CI 1.32–1.40). Persons identifying as American Indian/Alaska Native had a higher rate of suspected reinfection (53/1,000 cases) than other racial and ethnic groups; persons identifying as Hispanic had rates of 48/1,000 cases, persons identifying as multiracial had 43/1,000 cases, and persons identifying as non-Hispanic Black had 40/1,000 cases. We observed lower suspected reinfection rates among persons identifying as Asian or Pacific Islander (38/1,000 cases) and non-Hispanic White (35/1,000 cases). We observed higher odds of suspected reinfection among all non-White racial and ethnic groups compared with non-Hispanic White, but we did not see statistically significant differences among persons identifying as multiracial (OR 1.26, 95% CI 0.98–1.62; p = 0.077) (Table).

**Table:** Characteristics of persons with suspected SARS-CoV-2 reinfections, Clark County, Nevada, USA, December 2021

Characteristics	Suspected reinfection rate per 1,000 primary cases	Odds ratio (95% CI)	p value
Age group, y			
0–4	15	0.30 (0.26–0.35)	<0.0001
5–17	29	0.58 (0.55–0.62)	<0.0001
18–24	49	Referent	Referent
25–49	46	0.95 (0.91–0.99)	0.0246
50–64	33	0.66 (0.63–0.69)	<0.0001
>65 years	21	0.42 (0.40–0.45)	<0.0001
Sex			
F	44	1.36 (1.32–1.40)	<0.0001
M	33	Referent	Referent
Race/ethnicity*			
American Indian/Alaska Native	53	1.55 (1.10–2.19)	0.0116
Asian or Pacific Islander	38	1.08 (1.02–1.14)	0.0087
Black	40	1.17 (1.10–1.23)	<0.0001
Hispanic	48	1.40 (1.34–1.45)	<0.0001
Multiracial	43	1.26 (0.98–1.62)	0.0771
White	35	Referent	Referent

*Hispanic persons could be of any race; American Indian/Alaska Native, Asian or Pacific Islander, Black, White, and multiracial persons were non-Hispanic.

**Figure 2:**
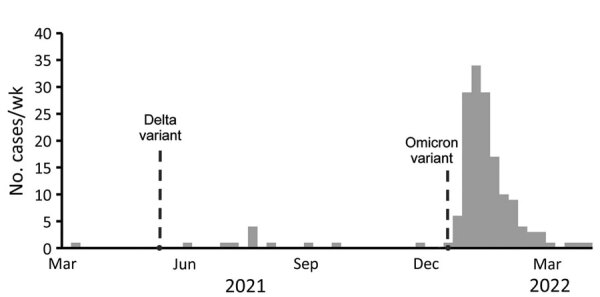
Suspected third SARS-CoV-2 infections, Clark County, Nevada, USA, March 2021–March 2022. Dotted lines show timeframe for identification of Delta and Omicron SARS-CoV-2 variants in Clark County. Suspected third infections were defined as a third positive PCR test collected >90 days after a second positive test collected >90 days after a person’s initial positive PCR test.

From the beginning of March 2021 through April 2, 2022, we identified 161 suspected third infections among Clark County residents. Thirteen of those infections occurred sporadically from March 2021 through the week of December 12–18, 2021. Beginning the week of December 19–25, the rate rapidly increased, and 92% (148/161) of the suspected third infections occurred after the Omicron variant was detected. The number of suspected third infections declined during January–March 2022, mirroring trends in primary infections and suspected reinfections (Figure 2).

## Discussion

This population-level analysis shows that suspected SARS-CoV-2 reinfections were relatively rare in Clark County before the Omicron variant was detected but that suspected reinfection accounted for a substantially higher proportion of COVID-19 cases during December 2021–March 2022. Although the weekly number of suspected reinfections fell in concert with the number of primary cases, the proportion of all cases that were suspected reinfections remained around peak levels of 11%. This trend is consistent with reports from other jurisdictions (*7*) and supports the hypothesis that prior infection might be less protective against infection with the Omicron variant than against previous SARS-CoV-2 variants (*1*,*8*). Suspected third infections were rare in Clark County but increased during December 2021 after the Omicron variant was detected, suggesting multiple SARS-CoV-2 infections might occur more frequently as genetically diverse variants are introduced.

Among previously infected persons, the incidence of suspected reinfection was highest among adults <50 years of age, women, and persons identifying as American Indian/Alaska Native. We also observed elevated incidence among persons identifying as Hispanic, multiracial, non-Hispanic Black, and Asian or Pacific Islander compared with persons identifying as non-Hispanic White. This finding might indicate a higher risk for repeated exposure among these groups. These disparities mirror disparities observed among primary cases in Clark County, except for a comparatively low rate of primary infection observed among persons identifying as American Indian/Alaska Native (*9*) but a comparatively high rate of suspected reinfection among this group. This discrepancy is notable; however, race and ethnicity data were missing for ≈26% of persons with primary infections and 21% of persons with suspected reinfection. We do not know whether this finding would persist if the data were more complete.

The first limitation of our study is that we only examined suspected reinfections meeting the 90-day interval criteria. We did not confirm reinfection by using viral genomic sequencing or other data, such as known COVID-19 exposure before a second occurrence or negative test between occurrences. We did not identify reinfection cases occurring within the 90-day interval, regardless of viral genomic sequencing or clinical suspicion of reinfection. Second, we did not match viral lineage data to specimens from suspected reinfections, so the rapid increase in suspected cases cannot definitively be linked to the Omicron variant. However, by the week of December 19, 2021, 79% of sequenced samples from Clark County were the Omicron variant, and that proportion continued to increase through January 2022 (*10*). Finally, the large proportion of missing race and ethnicity data adds uncertainty to findings regarding the racial and ethnic groups most affected. By comparison, age and sex data were missing for <1% of persons. Some occupational or demographic groups, such as healthcare workers and college students, might undergo COVID-19 screening more frequently than others (*11*), which could lead to a disproportionate reduction of missed diagnoses among some groups and affect incidence estimates of primary infections and suspected reinfections.

Additional data of interest, including vaccination status, prior health status, and illness severity, were not available for this analysis. Other studies have observed reduced risk for severe illness during suspected reinfection compared with primary infection (*12*,*13*). As with primary infection, older age, male sex, and comorbidities have been described as risk factors for severe outcomes during reinfection (*12*).

COVID-19 vaccination has been associated with reduced risk for reinfection (*14*) and reduced risk for intensive care unit admission during reinfection (*12*). By the end of March 2022, ≈91% of Nevada residents >70 years of age had initiated COVID-19 vaccination, more than any other age group in the state (*15*). Vaccination might contribute to the relatively lower rates of suspected reinfection we observed among older persons in Clark County during this investigation. However, children <5 years of age were not eligible for COVID-19 vaccination until June 2022 (*16*), so vaccination rates do not explain the relatively lower rates of suspected reinfection we observed among young children. The proportion of other adult groups initiating vaccination in Nevada ranged from ≈61% for persons 20–29 years of age to ≈84% for persons 60–69 years of age (*15*). During the same timeframe, ≈54% of Nevada residents identifying as Asian or Pacific Islander had initiated COVID-19 vaccination, as had ≈53% of residents identifying as Hispanic and ≈44% of those identifying as non-Hispanic White (*15*). Vaccination initiation was lower among persons identifying as non-Hispanic Black (≈40%) and American Indian or Alaska Native (33%) (*15*). Lagging vaccination rates among racial and ethnic minority groups could be a contributing factor to the higher rates of suspected reinfection observed among these groups. However, the higher rates of vaccine initiation among Nevada residents and higher rates of suspected reinfection among Clark County residents identifying as Asian or Pacific Islander and Hispanic compared with those identifying as non-Hispanic White suggest a different factor driving risk for reinfection among these groups. 

Reduced vaccine effectiveness (VE) against COVID-19–associated emergency department and urgent care encounters and hospitalizations has been observed during the Omicron wave compared with the Delta wave (*17*). However, VE against hospitalization was still high during the Omicron wave, especially among persons who received a second vaccine dose within 180 days before the healthcare encounter (VE 81%, 95% CI 65%–90%) and persons who received a third dose (VE 90%, 95% CI 80%–94%) (*17*). In our analysis, we did not know whether persons with suspected reinfection were vaccinated, the interval between vaccination and second positive test, or whether illness severity during reinfection was different between vaccinated and unvaccinated persons. Vaccination status is only one of many factors, including occupational and social exposures, that likely influence risk for reinfection.

Although formerly rare, suspected SARS-CoV-2 reinfection has occurred more frequently in Clark County after the emergence of the Omicron variant. Several factors might contribute to reduced protection against repeated infection, including the emergence of variants capable of immune evasion and waning immunity as the interval from initial infection or vaccination increases for many persons (*18*,*19*). Some demographic groups are disproportionately affected by suspected reinfection in Clark County. Further investigation into factors driving these disparities could inform prevention measures. Further investigation also might help determine impacts of these disparities, such as the burden of severe disease, the economic burden of repeated isolation and quarantine, and whether the same disparities are observed in other jurisdictions. Public health surveillance is necessary to clarify the burden of SARS-CoV-2 reinfection in communities. COVID-19 vaccination is one essential tool to help prevent SARS-CoV-2 reinfection and reduce disease severity when reinfection occurs, and vaccination efforts could help curb reinfection risk.
